# A Novel Multiplex PCR Discriminates *Bacillus anthracis* and Its Genetically Related Strains from Other *Bacillus cereus* Group Species

**DOI:** 10.1371/journal.pone.0122004

**Published:** 2015-03-16

**Authors:** Hirohito Ogawa, Daisuke Fujikura, Miyuki Ohnuma, Naomi Ohnishi, Bernard M. Hang'ombe, Hitomi Mimuro, Takayuki Ezaki, Aaron S. Mweene, Hideaki Higashi

**Affiliations:** 1 Hokudai Center for Zoonosis Control in Zambia, School of Veterinary Medicine, The University of Zambia, Lusaka, Zambia; 2 Department of Disease Control, School of Veterinary Medicine, The University of Zambia, Lusaka, Zambia; 3 Division of Infection and Immunity, Research Center for Zoonosis Control, Hokkaido University, Sapporo, Japan; 4 Department of Paraclinical Studies, School of Veterinary Medicine, The University of Zambia, Lusaka, Zambia; 5 Division of Bacteriology, Department of Infectious Diseases Control, International Research Center for Infectious Diseases, Institute of Medical Science, The University of Tokyo, Tokyo, Japan; 6 Pathogenic Microbes Repository Unit, International Research Center for Infectious Diseases, Institute of Medical Science, The University of Tokyo, Tokyo, Japan; 7 Department of Microbiology, Regeneration and Advanced Medical Science, Gifu University Graduate School of Medicine, Gifu, Japan; 8 Global Station for Zoonosis Control, Global Institution for Collaborative Research and Education, Hokkaido University, Sapporo, Japan; INIAV, I.P.- National Institute of Agriculture and Veterinary Research, PORTUGAL

## Abstract

Anthrax is an important zoonotic disease worldwide that is caused by *Bacillus anthracis*, a spore-forming pathogenic bacterium. A rapid and sensitive method to detect *B*. *anthracis* is important for anthrax risk management and control in animal cases to address public health issues. However, it has recently become difficult to identify *B*. *anthracis* by using previously reported molecular-based methods because of the emergence of *B*. *cereus*, which causes severe extra-intestinal infection, as well as the human pathogenic *B*. *thuringiensis*, both of which are genetically related to *B*. *anthracis*. The close genetic relation of chromosomal backgrounds has led to complexity of molecular-based diagnosis. In this study, we established a *B*. *anthracis* multiplex PCR that can screen for the presence of *B*. *anthracis* virulent plasmids and differentiate *B*. *anthracis* and its genetically related strains from other *B*. *cereus* group species. Six sets of primers targeting a chromosome of *B*. *anthracis* and *B*. *anthracis*-like strains, two virulent plasmids, pXO1 and pXO2, a bacterial gene, 16S rRNA gene, and a mammalian gene, actin-beta gene, were designed. The multiplex PCR detected approximately 3.0 CFU of *B*. *anthracis* DNA per PCR reaction and was sensitive to *B*. *anthracis*. The internal control primers also detected all bacterial and mammalian DNAs examined, indicating the practical applicability of this assay as it enables monitoring of appropriate amplification. The assay was also applied for detection of clinical strains genetically related to *B*. *anthracis*, which were *B*. *cereus* strains isolated from outbreaks of hospital infections in Japan, and field strains isolated in Zambia, and the assay differentiated *B*. *anthracis* and its genetically related strains from other *B*. *cereus* group strains. Taken together, the results indicate that the newly developed multiplex PCR is a sensitive and practical method for detecting *B*. *anthracis*.

## Introduction

Anthrax is an important worldwide zoonosis caused by the spore-forming bacterium *Bacillus anthracis*, a Gram-positive, rod-shaped bacterium that is transmitted among soil, wildlife, livestock and humans. This disease is still enzootic in most countries in Africa and Asia and occurs sporadically in Europe, the American continent and Australia [1]. The cycle of infection is primarily found in herbivores (e.g., cattle, sheep, goats) infected after contact with soil contaminated with spores. Spores ingested by herbivores germinate within the host to produce vegetative forms, which multiply and express their virulence factors, killing the host [2]. Human infections are caused by contact with infected animals or their products, and the following three forms of anthrax are found depending on the three different infection routes: cutaneous, gastrointestinal and inhalation forms of anthrax. Cutaneous anthrax accounts for more than 95% of human cases worldwide [3]. In cases of gastrointestinal anthrax, the lesion may occur anywhere within the gastrointestinal tract, though mostly in the ileum and cecum, after ingestion of contaminated meat that has not been sufficiently cooked. In recent years, injection anthrax has been identified as the fourth form of anthrax [4]. This type of anthrax is recognized among the drug (e.g., heroin) users in Europe, and the symptoms depend on the type of infection (i.e., cutaneous, gastrointestinal, and inhalation).

*B*. *anthracis* is an important pathogen that has been extensively studied not only for maintenance of public health worldwide but also for the defense against an effective biological weapon. Indeed, envelopes containing *B*. *anthracis* spores were mailed to news media companies and government officials, leading to the first bioterrorism-related cases of anthrax in the United States of America in 2001 [5]. Anthrax occurred in 22 people with 5 deaths from inhalation anthrax in these biological attacks. The lethal dose for 50% of test subjects for humans is between 8,000 and 10,000 spores, and once symptoms of inhalational anthrax appear, treatment is almost invariably ineffective [6].

A rapid and sensitive method to detect *B*. *anthracis* is important for control of anthrax in animal cases to maintain public health and for appropriate treatment in human cases. Several PCR [1,7–9], real-time PCR [10] and loop-mediated isothermal amplification (LAMP) [11] techniques have been developed to detect chromosomal genes (e.g., S-layer (*sap*) and Ba813), virulent plasmid pXO1, encoding protective antigen (*pag*), lethal factor (*lef*) and edema factor (*cya*), and virulent plasmid pXO2, encoding capsule protein (*cap*). These genes have been used as markers to discriminate *B*. *anthracis* from other bacteria in the *B*. *cereus* group, which includes *B*. *anthracis*, *B*. *cereus* (known as a potential food poisoning pathogen) [12], *B*. *thuringiensis* (known as an insect pathogen) [13], *B*. *mycoides*, *B*. *pseudomycoides* and *B*. *weihenstephanensis*. Among the *B*. *cereus* group species, *B*. *anthracis*, *B*. *cereus* and *B*. *thuringiensis* are considered as the same species based on genetic evidence [14] and are difficult to discriminate despite the existence of gene markers. Furthermore, there have been reports about severe manifestations and fatal cases caused by *B*. *cereus* (extra-intestinal *B*. *cereus*, e.g., strains F837/76, 03BB102) [15,16], *B*. *thuringiensis* isolated from a necrotic human wound (human pathogenic *B*. *thuringiensis*, e.g., 97–27 subsp. *konkukian* serotype H34) [17,18] and *B*. *anthracis*-like strains (e.g., *B*. *cereus* var. anthracis strain CI isolated from chimpanzees) [19,20]. Some of these bacteria have been shown to harbor *B*. *anthracis* virulent plasmids [21–23] or had chromosomal backgrounds closely related to *B*. *anthracis* [16,17]. Therefore, the establishment of a new detection method is needed to discriminate *B*. *anthracis* from other *B*. *cereus* group species including the above genetically related strains of *B*. *anthracis*.

In this study, we developed a practical *B*. *anthracis* multiplex PCR containing internal control primers targeting exogenous bacterial and mammalian genes. This assay is capable of screening for the presence of pXO1 and pXO2 and of differentiating *B*. *anthracis* and its genetically related strains from other *B*. *cereus* group.

## Materials and Methods

### Bacterial strains and culture conditions

*Bacillus* strains and bacterial DNAs used for optimization of *16S rRNA* amplification are listed in Tables 1–3. *Bacillus* species listed in Tables 1 and 2, excepted for Sterne 34F2, GTC02891, GTC02896, GTC02916, GTC02917, GTC03222 and GTC02826T, were isolated from natural sources according to the following protocol. One gram of specimen was suspended in 10 ml of sterilized saline followed by incubation at 75°C for 20 min. An aliquot of the sample was inoculated on 10% (v/v) sheep blood agar and cultured at 37°C overnight. The isolates were used in this study after cloning and characterization. *Bacillus* strains were grown on brain heart infusion agar or 10% (v/v) sheep blood agar at 37°C overnight for subsequent studies. *B*. *anthracis* was handled in the biosafety level 3 facility of the Hokudai Center for Zoonosis Control in Zambia, School of Veterinary Medicine, The University of Zambia. Six GTC strains were obtained from the Pathogenic Microorganism Genetic Resource Stock Center, School of Medicine, Gifu University (Table 2). Thirteen bacterial genomes were obtained from the Pathogenic Microbes Repository Unit, International Research Center for Infectious Diseases, Institute of Medical Science, The University of Tokyo (Table 3).

**Table 1:** *Bacillus anthracis* strains used in this study.

Species	Strain	Hemolysis^1^	Plasmid Content		BA_5031^2^	Source or reference
			*pag*	*cap*		
*Bacillus anthracis*	CZC5	Non-hemolytic	+	+	+	Isolated from hippopotamus in the Luangwa valley Zambia, 2011 [28]
*Bacillus anthracis*	CZC5 clone1	Non-hemolytic	-	+	+	Laboratory strain modified from the strain CZC5
*Bacillus anthracis*	Sterne 34F2	Non-hemolytic	+	-	+	Commercial vaccine strain
*Bacillus anthracis*	Sterne 34F2 clone2	Non-hemolytic	-	-	+	Laboratory strain modified from the strain Sterne 34F2
*Bacillus anthracis*	M1	Non-hemolytic	+	+	+	Isolated from cattle in Mongu, Zambia, 2012
*Bacillus anthracis*	L158–1	Non-hemolytic	+	+	+	Isolated from soil in the Lower Zambezi, Zambia, 2012

^1^Hemolysis was examined on 10% (v/v) sheep blood agar.

^2^BA_5031, tentatively termed from the locus tag name of *Bacillus anthracis* strain Ames, indicates Ba813 region.

**Table 2:** Other *Bacillus* strains used in this study.

Species	Strain	Hemolysis^1^	Plasmid Content		BA_5031^2^	Source or reference
			*pag*	*cap*		
*Bacillus cereus*	LZ48–5	Hemolytic	-	-	+	Isolated from soil in the Lower Zambezi, Zambia, 2012
*Bacillus cereus*	LZ136–1	Hemolytic	-	-	+	Isolated from baboon carcass in the Lower Zambezi, Zambia, 2012
*Bacillus cereus*	LZ136–2	Hemolytic	-	-	+	Isolated from baboon carcass in the Lower Zambezi, Zambia, 2012
*Bacillus cereus*	LZ77–1	Non-hemolytic^3^	-	-	+	Isolated from soil in the Lower Zambezi, Zambia, 2012
*Bacillus cereus*	LZ77–2	Non-hemolytic^3^	-	-	+	Isolated from soil in the Lower Zambezi, Zambia, 2012
*Bacillus cereus*	LZ78–7	Non-hemolytic^3^	-	-	+	Isolated from soil in the Lower Zambezi, Zambia, 2012
*Bacillus cereus*	LZ78–8	Non-hemolytic^3^	-	-	+	Isolated from soil in the Lower Zambezi, Zambia, 2012
*Bacillus cereus*	GTC02891	Hemolytic	-	+	+	Isolated from independent human blood cultures at Gifu University hospital [23]
*Bacillus cereus*	GTC02896	Hemolytic	-	-	+	Isolated from independent human blood cultures at Gifu University hospital [23]
*Bacillus cereus*	GTC02916	Hemolytic	-	-	+	Isolated from independent human blood cultures at Gifu University hospital [23]
*Bacillus cereus*	GTC02917	Hemolytic	-	-	+	Isolated from independent human blood cultures at Gifu University hospital [23]
*Bacillus cereus*	GTC03222	Hemolytic	-	-	+	Isolated from independent human blood cultures at Jichi Medical University [23]
*Bacillus cereus*	GTC02826T	Hemolytic	-	-	-	JCM 2152 [23]
*Bacillus cereus*	BC_CZC1	Hemolytic	-	-	-	Isolated from soil in Hokkaido, Japan, 2014
*Bacillus cereus*	BC_CZC2	Hemolytic	-	-	-	Isolated from soil in Hokkaido, Japan, 2014
*Bacillus pseudomycoides*	BP_CZC1	Hemolytic	-	-	-	Isolated from soil in Hokkaido, Japan, 2014
*Bacillus pseudomycoides*	BP_CZC2	Hemolytic	-	-	-	Isolated from soil in Hokkaido, Japan, 2014

^1^Hemolysis activities on 10% (v/v) sheep blood agar were examined at 35°C for 24 hrs.

^2^BA_5031, tentatively termed from the locus tag name of *Bacillus anthracis* strain Ames, indicates Ba813 region.

^3^After incubation at 35°C for 48 hrs, weak hemolytic activity was displayed.

**Table 3:** Bacterial DNAs used for optimization of *16S rRNA* amplification in this study.

Species		Administration No.^1^	Morphotype	Reference
**Gram-positive bacteria**				
	*Bacillus cereus*	IID1681	Bacillus	ATCC 14579
	*Bacillus megaterium*	IID928	Bacillus	IAM 1166
	*Bacillus subtilis*	IID864	Bacillus	ATCC 6633
	*Clostridiun perfringens*	IID520	Bacillus	ATCC 10543
	*Clostridiun tetani*	IID524	Bacillus	IID RIKU3
	*Lactobacillus acidophilus*	IID892	Bacillus	ATCC 393
	*Listeria monocytogenes*	IID577	Bacillus	ATCC 19111
**Gram-negative bacteria**				
	*Staphylococcus aureus*	IID671	Coccus	FDA 209P
	*Staphylococcus epidermidis*	IID866	Coccus	ATCC 12228
	*Salmonella enterica* subsp. *enterica* serovar Choleraesuis	IID1682	Bacillus	ATCC 13312
	*Shigella boydii*	IID627	Bacillus	NIHJ 1130
	*Vibrio cholerae*	IID3006	Bacillus	HAKATA 487–85
	*Campylobactoer jejuni*	IID1654	Spirillum	819–481

^1^Administration No. was numbered in Pathogenic Microbes Repository Unit, International Research Center for Infectious Diseases, Institute of Medical Science, The University of Tokyo.

**Fig 1:**
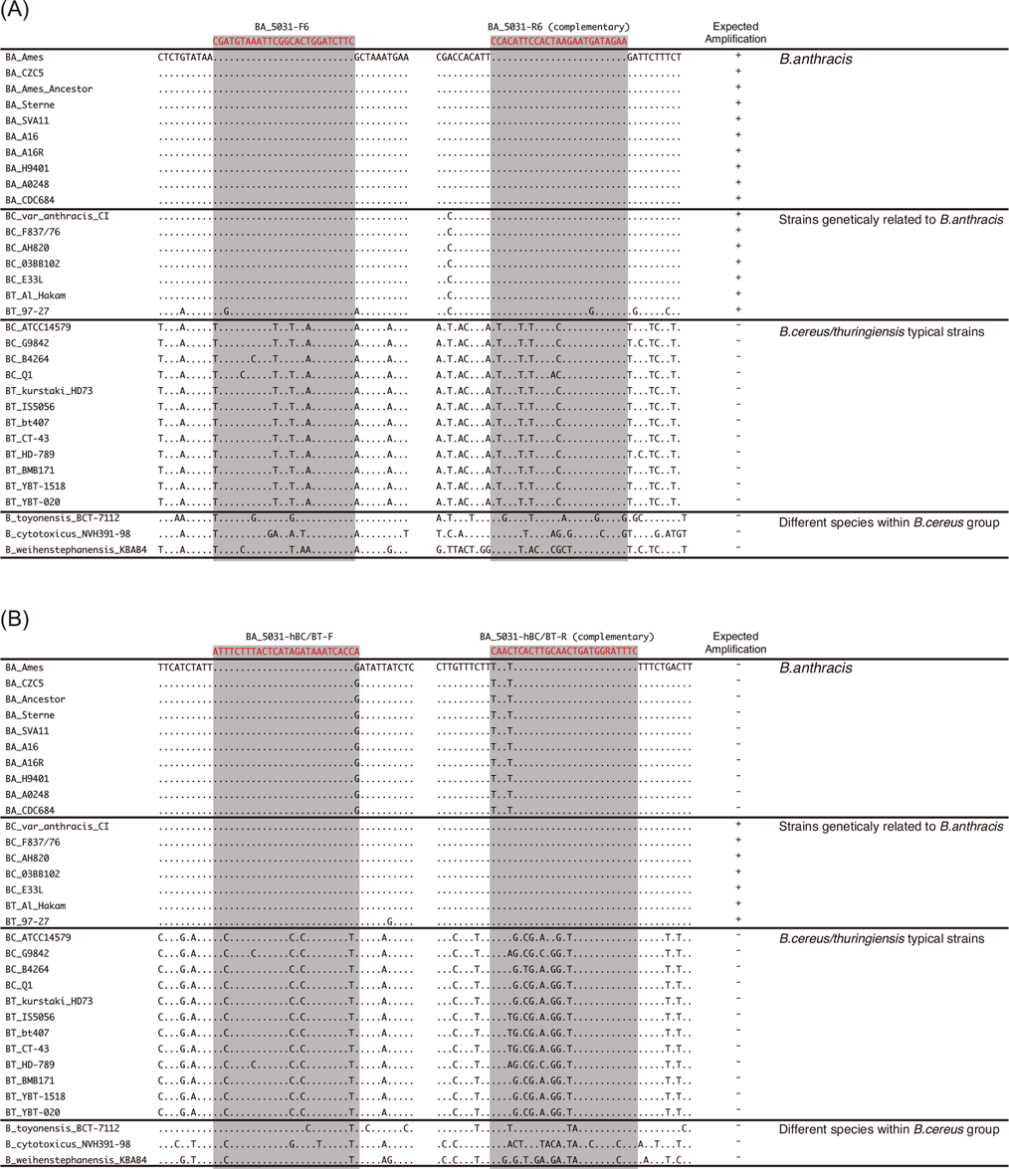
BA_5031 and hBC/BT primer annealing sites on genome sequences of *Bacillus cereus* group strains. Alignment of *B*. *anthracis*, *B*. *cereus*, *B*.*thuringiensis*, *B*. *toyonensis*, *B*. *cytotoxis* and *B*. *weihenstephanensis* BA_5031 (A) and BACI_c47770 (B) sequences from the database and primer sequences in this study. The rectangles shaded with grey correspond to BA_5031 and BACI_c47770 primer sequences. GENETYX-MAC Network Version 15.0.5 software was used for multiple sequence alignment. Expected amplification was simulated *in silico* using Amplifx 1.7.0 software.

In this study, five GTC strains (GTC02891, GTC02896, GTC02916, GTC02917 and GTC03222) shown in Table 2 as well as *B*. *cereus* var. anthracis strain CI, four *B*. *cereus* strains (F837/76, AH820, 03BB102 and E33L) and two *B*. *thuringiensis* strains (97–27 subsp. *konkukian* serotype H34 and Al Hakam) shown in Fig. 1 were used as genetically related strains of *B*. *anthracis*.

### Human and animal samples

Blood or tissue samples from a human (*Homo sapiens*) and 13 animals were used in this study. The animals included cattle (*Bos taurus*), sheep (*Ovis aries*), goat (*Capra hircus*), donkey (*Equus asinus*), rabbit (*Oryctolagus cuniculus*), African elephant (*Loxodonta africana*), African buffalo (*Syncerus caffer*), hippopotamus (*Hippopotamus amphibius*), puku (*Kobus vardonii*), warthog (*Phacochoerus africanus*), spotted hyena (*Crocuta crocuta*), bushbuck (*Tragelaphus scriptus*), and lion (*Panthera leo*), of which 8 wildlife animals were from South Luangwa National Park, Lower Zambezi National Park, and their game management areas.

### Ethical statements for the project

Approval for wildlife animal sampling and soil sampling in the national park and game management areas was obtained from the Zambia Wildlife Authority (ZAWA) in the Republic of Zambia. Some of the animals were hunted by a hunting company for meat with permission from ZAWA to control their large numbers, and tissue specimens from non-endangered species, African buffalo, puku, warthog, spotted hyena and bushbuck, were kindly provided. Other samples were taken from carcasses to determine the cause of death. We received a waiver of approval from the University of Zambia Research Ethics Committee to do animal research following approval from ZAWA and the Ministry of Agriculture and Livestock. The human blood sample was taken from the first author (Dr. Hirohito Ogawa) with the author’s consent.

### DNA extraction

A bacteria colony on the agar plate was suspended in 100 μl of sterile phosphate buffered saline followed by heating at 95°C for 15 min and centrifugation at 15,000 rpm for 2 min at 4°C, and then 1 μl of the supernatant was directly used for PCR.

Human and animal DNAs were extracted from 100 μl of blood or 10% (w/v) tissue homogenate by using TRIzol Reagent (Life Technologies) or QIAamp DNA Mini Kit (QIAGEN) according to the manufacturer’s instructions. The extracted DNA was dissolved in 100 μl of distilled water or Buffer AE (QIAGEN).

For a sensitivity test, 100 μl of LB broth was collected, followed by heating at 95°C for 20 min and centrifugation at 15,000 rpm for 2 min at 4°C, and then 1 μl of the supernatant was directly used for PCR.

### Primer design

Six primer sets were designed for the multiplex PCR targeting the *B*. *anthracis* chromosome and two plasmids, pXO1 and pXO2, common bacterial and mammalian genes and chromosome of the genetically related strains of *B*. *anthracis* to detect and discriminate *B*. *anthracis* and other *Bacillus* species by the amplified band patterns (Table 4). Three primer sets targeting the *B*. *anthracis* Ba813 region, tentatively termed “BA_5031” from the locus tag name of *B*. *anthracis* strain Ames, on the chromosome, *pag* encoded on pXO1 and *cap* encoded on pXO2 were designed to detect *B*. *anthracis* genes. One primer set (hBC/BT) targeting the region, tentatively termed “BACI_c47770” from the locus tag name of *Bacillus cereus* var. anthracis strain CI [19], which was capable of detecting the region of the genetically related strains of *B*. *anthracis* but not that of *B*. *anthracis*, was designed for discriminating them. Two primer sets targeting the 16S rRNA gene (*16S rRNA*) on a bacterial chromosome and beta-actin gene (*ACTB*) on a mammalian chromosome were designed as internal controls. Amplifx 1.7.0 (http://crn2m.univ-mrs.fr/pub/amplifx-dist) was used to simulate PCR reaction using the newly designed primers.

**Table 4:** Primers used in this study.

Primer	Sequence (5’-3’)	Target gene	Product size	Reference (Accession No. ^1^)
BA_5031-F6	CGATGTAAATTCGGCACTGGATCTTC	BA_5031	1027 bp	*Bacillus anthracis* strain Ames (AE016879)
BA_5031-R6	TTCTATCATTCTTAGTGGAATGTGG			
16S-663F	AKGTGTAGCGGTGAAATGCGTAG	*16S rRNA*	733 bp	*Bacillus anthracis* strain Ames *16S ribosomal RNA* gene (NR_074453)
16S-1395R	TGGTGTGACGGGCGGTGTGTACAAGG			
CAP-F8	TCATCCGGATCCAGGAGCAATGAG	*cap*	578 bp	*Bacillus anthracis* strain TE702 *encapsulation protein* gene (M24150)
CAP-R7	GCAGGTAAAATACCTGTTCTTTCTG			
PA-F6	CCTTGTGGCAGCTTATCCGA	*pag*	364 bp	*Bacillus anthracis* strain Sterne plasmid pX01 (NC_001496)
PA-R6	GTAGATTGGAGCCGTCCCAG			
ACTB-F3813	CAGATCATGTTYGAGACCTTCAACAC	*ACTB*	224 bp	*Homo sapiens actin*, *beta* gene (AY582799)
ACTB-R4036	TCVGTSAGGATCTTCATGAGGTAGTC			
hBC/BT-F	ATTTCTTTACTCATAGATAAATCACCA	BACI_c47770	197 bp	*Bacillus cereus* var. anthracis strain CI (CP001746)
hBC/BT-R	GAAATYCCATCAGTTGCAAGTGAGTTG			

^1^Accession No. indicates Genbank accession number of reference sequence.

### PCR with newly designed primer set(s)

Multiplex PCR was carried out using a TaKaRa Multiplex PCR Assay Kit (TaKaRa) according to the manufacturer’s instructions. A total volume of 25 μl of reaction mixture containing 0.6 μM of BA_5031 primer set, 0.2 μM of *cap* and *ACTB* primer sets, 0.1 μM of hBC/BT primer set, 0.05 μM of *16S rRNA* and *pag* primer sets and 1 μl of template DNA was used (Table 4). The multiplex PCR program consisted of initial denaturation at 94°C for 1 min followed by 35 cycles of denaturation at 94°C for 30 sec, annealing at 58°C for 2 min, extension at 72°C for 2 min, and final extension at 72°C for 10 min.

A PCR using each primer set was carried out using a TaKaRa Multiplex PCR Assay Kit (TaKaRa) according to the manufacturer’s instructions. A total volume of 25 μl of reaction mixture containing 0.2 μM of each primer and 1 μl of template DNA was used. The PCR program consisted of initial denaturation at 94°C for 30 sec followed by 35 cycles of denaturation at 94°C for 30 sec, annealing at 58°C for 25 sec, and extension at 72°C for 1 min.

### Previously reported PCR compared to the newly developed multiplex PCR

Five PCRs with each primer shown in Table 5 were carried out using a TaKaRa Multiplex PCR Assay Kit (TaKaRa) according to the manufacturer’s instructions. Primer concentration and annealing temperature were the same as those in previous studies [1,7–10]. The PCR program for single PCR consisted of initial denaturation at 94°C for 1 min followed by 35 cycles of denaturation at 94°C for 30 sec, annealing at a stable temperature for each primer set (Table 4) for 25 sec, extension at 72°C for 1 min, and final extension at 72°C for 5 min. The PCR program for duplex PCR consisted of initial denaturation at 94°C for 1 min followed by 35 cycles of denaturation at 94°C for 30 sec, annealing at a stable temperature for primer sets (Table 4) for 90 sec, extension at 72°C for 90 sec, and final extension at 72°C for 5 min.

**Table 5:** PCR using primers designed in previous studies.

PCR						
	Primer	Sequence (5’-3’)	Target gene	Annealing temp	Product size	Reference
Duplex PCR to detect pXO1 and pXO2 plasmids recommended in WHO^1^ guideline						
	CAP1234	CTGAGCCATTAATCGATATG	*cap*	55°C	846 bp	[9]
	CAP1301	TCCCACTTACGTAATCTGAG				
	PA5	TCCTAACACTAACGAAGTCG	*pag*	55°C	596 bp	[7]
	PA8	GAGGTAGAAGGATATACGGT				
PCR to detect *Bacillus anthracis* chromosome recommended in WHO guideline						
	S-layer Upper	CGCGTTTCTATGGCATCTCTTCT	*sap*	55°C	639 bp	[1]
	S-layer Lower	TTCTGAAGCTGGCGTTACAAAT				
Duplex PCR published by Brightwell *et al*.						
	CAP1	TAGGAGTTACACTGAGCC	*cap*	56°C	341 bp	[8]
	CAP2	AATGGTAACCCTTGTCTTTG				
	BA813 R1	TTAATTCACTTGCAACTGATGGG	Ba813	56°C	152 bp	[8]
	BA813 R2	AACGATAGCTCCTACATTTGGAG				
PCR to detect pXO2 published by Makino *et al*.						
	MO11	GACGGATTATGGTGCTAAG	*cap*	60°C	591 bp	[10]
	MO12	GCACTGGCAACTGGTTTTG				
PCR to detect pXO1 published by Beyer *et al*.						
	PA7	ATCACCAGAGGCAAGACACCC	*pag*	60°C	211 bp	[7]
	PA6	ACCAATATCAAAGAACGACGC				
TaKaRa *Bacillus anthracis* PCR Detection Kit (RR027)						
	MO11	GACGGATTATGGTGCTAAG	*cap*	60°C	591 bp	[10]
	MO12	GCACTGGCAACTGGTTTTG				
	PA7	ATCACCAGAGGCAAGACACCC	*pag*	60°C	211 bp	[7]
	PA6	ACCAATATCAAAGAACGACGC				

^1^WHO, World Health Organization.

A commercial kit (TaKaRa *Bacillus anthracis* PCR Detection Kit, TaKaRa) was used according to the manufacturer’s instructions. This PCR system contained internal control genes for *pag* and *cap*. Either or both internal control bands amplified by the *pag* primer set (409 bp) and *cap* primer set (98 bp) would appear when the detection limit for *pag* and *cap* was below or the sample was lacking either or both of the plasmids.

### Sensitivity test of multiplex PCR

The *B*. *anthracis* strain CZC5 (Accession number: BAVT00000000) [24] was cultured on LB agar at 37°C overnight. One colony with a diameter of approximately 3 mm was suspended in 3 ml of LB broth followed by serial 10-fold dilution in LB broth with 5% (v/v) sheep blood. Each 100 μl of suspension was collected from the dilution and used for DNA extraction according to the heating method described above. An aliquot from the dilution was cultured for counting colony-forming units (CFU/ml) on LB agar at 37°C overnight.

### Sequence analysis

Sequence analysis of the PCR product was carried out by direct sequencing or a cloning method using pGEM-T Easy Vector (Promega). Purified PCR products or plasmids were sequenced in a 3130xl Genetic Analyzer (Life Technologies) following reaction using a BigDye Terminator v3.1 Cycle Sequencing Kit (Life Technologies) according to the manufacturer’s instructions. T7-GG primer (5’-AATACGACTCACTATAGGG-3') and SP6 primer (5’-CAAGCTATTTAGGTGACACTATAG-3') were used for sequence analysis of the plasmid.

## Results

### Specificity of internal control primers targeting *ACTB* and *16S rRNA*

To confirm the host range of the *ACTB* primer set, we attempted to amplify *ACTB* from DNAs extracted from a human and various kinds of mammalian animals. As a result, all of them were amplified (S1 Fig.). Nucleotide sequences of all of the products were determined and identified as an *ACTB* derived from the respective templates.

Next, the *16S rRNA* primer set was assessed with a probe match function in Ribosomal Database Project (RDP) [25] *in silico* to determine the suitability for *16S rRNA* amplification in common bacteria. The 16S-663F and 16S-1395R obtained 93.72% and 95.67% coverage of 9209 strain type of the domain *Bacteria* sequences submitted to repositories, respectively, using RDP’s probe match function with three errors allowance in each primer under the following data set options: strain type, isolate source, all size, and good quality (S1 Table). Combined *in silico* performance of the 16S-663F and 16S-1395R set covered 90.15% of the domain *Bacteria* (S1 Table). As a practical validation, PCR using the 16S-663F and 16S-1395R primer set detected *16S rRNA* from *B*. *anthracis* genomic DNA and 13 bacterial genomic DNAs (Table 3) in two major bacteria phyla: *Proteobacteria* and *Firmicutes*.

### Specificity of primers targeting the chromosome and virulent plasmids of *B*. *anthracis*

For specific amplification of *B*. *anthracis*, four target genes, Ba813 region, tentatively termed “BA_5031” on the chromosome, *pag* on pXO1, *cap* on pXO2 and a chromosomal region tentatively termed “BACI_c47770”, which was a specific region for genetically related strains of *B*. *anthracis* (described in Materials and Methods), were designed (Table 4).

It is known that the Ba813, a 277-bp fragment encoded on *B*. *anthracis* chromosomal DNA, has been specific to *B*. *anthracis* [26]. However, some *B*. *cereus* strains harboring Ba813 have been isolated from humans with severe manifestations [15,21,27]. For amplification of chromosomal DNA, primers to amplify the 1027 bp of BA_5031 were designed for *B*. *anthracis* as well as the genetically related strains of *B*. *anthracis* (Fig. 1). Furthermore, we designed a hBC/BT primers to amplify the 197 bp of the region tentatively termed “BACI_c47770” for discriminating between *B*. *anthracis* and the genetically related strains of *B*. *anthracis* (Fig. 1). Each specific band was amplified by PCR using each primer set (S2 Fig.).

### Comparison of multiplex PCR and the existing PCR methods

**Fig 2:**
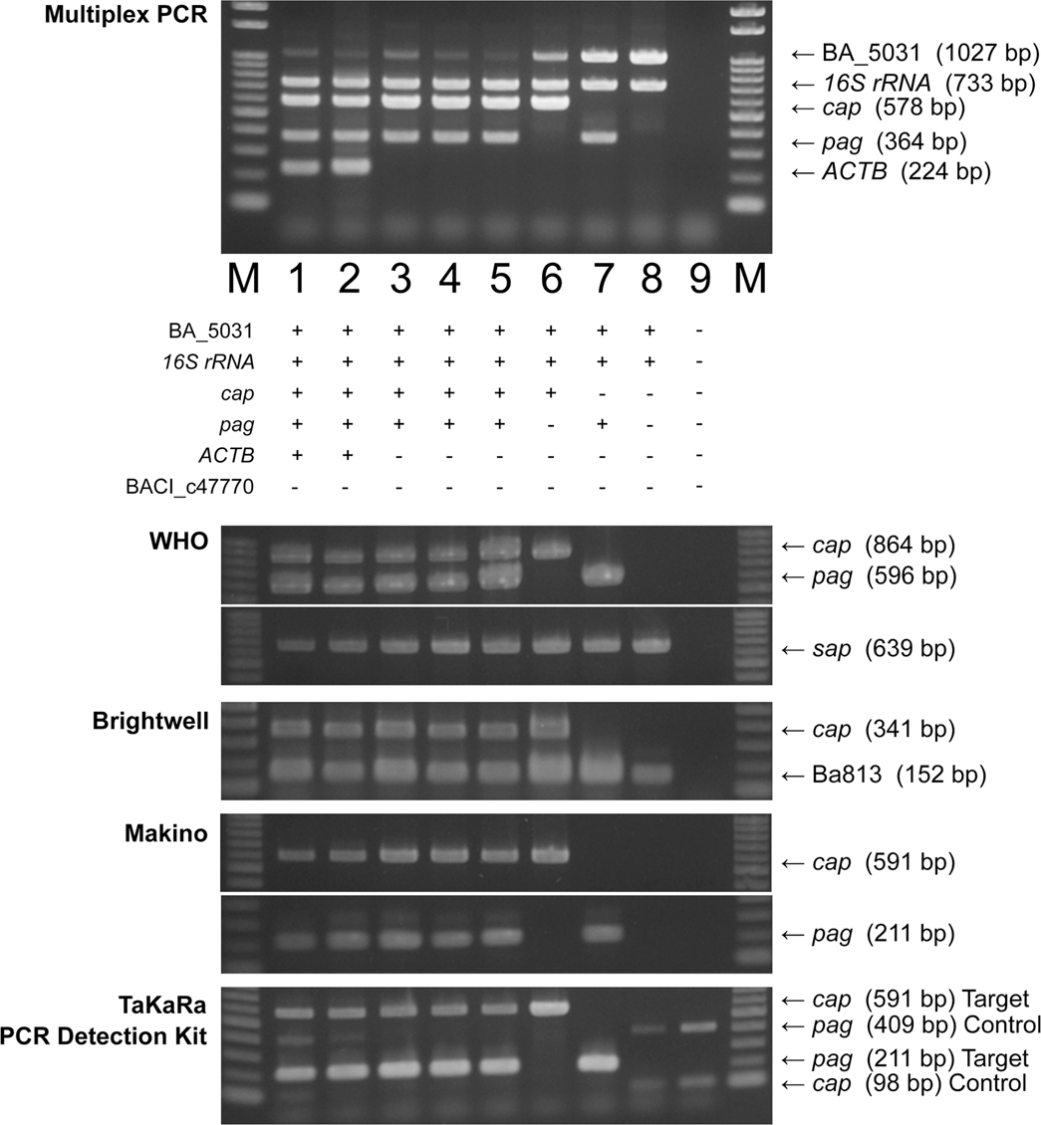
Comparison of the specificity of multiplex PCR and those of existing PCR methods. The expected DNA fragments corresponding to the characteristics of samples were amplified in all samples by the multiplex PCR assay established in this study (uppermost panel) and by three single PCRs, two duplex PCRs and PCR using a commercial kit (lower panels). Template DNA was extracted from *B*.*anthracis*-infected tissues and *B*.*anthracis* harboring or lacking virulent plasmids. Lane M, 100-bp DNA ladder; lane 1, DNA from *B*.*anthracis*-infected elephant blood; lane 2, DNA from *B*.*anthracis*-infected hippopotamus tissue; lane 3, DNA from *B*.*anthracis* strain CZC5; lane 4, DNA from *B*.*anthracis* strain M1; lane 5, DNA from *B*.*anthracis* strain L158–1; lane 6, DNA from *B*.*anthracis* strain CZC5 clone1; lane 7, DNA from *B*.*anthracis* vaccine strain Sterne 34F2; lane 8, *B*.*anthracis* vaccine strain Sterne 34F2 clone2; lane 9, distilled water as a negative control.

First, to examine whether the six newly designed primer sets could correctly amplify each target gene by the multiplex PCR simultaneously, we performed multiplex PCR for *B*. *anthracis*-infected elephant blood and hippopotamus tissue [28], *B*. *anthracis* isolates harboring both the virulent plasmids from a hippopotamus as a wildlife animal (strain CZC5: pXO1^+^/pXO2^+^/Ba813^+^/*16S rRNA* ^+^) [24,28], from a cow as a domestic animal (strain M1: pXO1^+^/pXO2^+^/Ba813^+^/*16S rRNA* ^+^) and from soil (strain L158–1: pXO1^+^/pXO2^+^/Ba813^+^/*16S rRNA* ^+^), *B*. *anthracis* strain CZC5 derivative lacking pXO1 (strain CZC5 clone1: pXO1^-^/pXO2^+^/Ba813^+^/*16S rRNA* ^+^), commercial animal vaccine strain lacking pXO2 (strain Sterne 34F2: pXO1^+^/pXO2^-^/Ba813^+^/*16S rRNA* ^+^) and *B*. *anthracis* strain Sterne 34F2 derivative lacking pXO1 (strain Sterne 34F2 clone2: pXO1^-^/pXO2^-^/Ba813^+^/*16S rRNA* ^+^). The expected fragments were amplified in all 8 samples (Fig. 2, uppermost panel).

Next, to compare the results, several PCR methods including World Health Organization (WHO) recommended methods [1,7–10] and a method using a commercial PCR kit were performed. These PCR methods also detected each gene according to the variation of the isolates (Fig. 2).

### Sensitivity of multiplex PCR using *B*. *anthracis* strain CZC5 in this study

**Fig 3:**
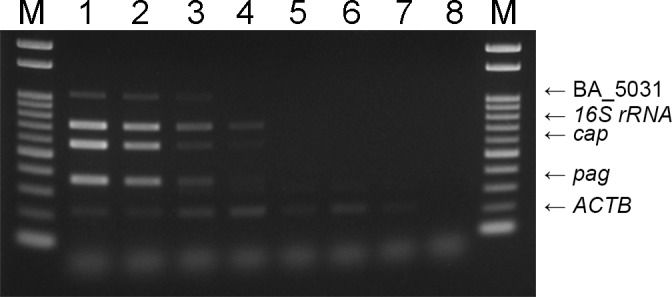
Sensitivity of multiplex PCR for *B*. *anthracis* cultured in liquid media. DNA fragments were amplified by the multiplex PCR in the extracted DNA from *B*. *anthracis* strain CZC5 cultured in LB broth containing 5% (v/v) sheep blood (lanes 1 to 7). All 5 bands were simultaneously detected at the template concentration of 2.99 × 10^3^ CFU/ml (lane 3). Lane M, 100-bp DNA ladder; lanes 1 to 7, serially 10-fold diluted *B*. *anthracis* strain CZC5. Lane 3, 2.99 × 10^3^ CFU/ml; lane 4, 3.83 × 10^2^ CFU/ml; lane 5, 2.0 × 10 CFU/ml; lanes 6 and 7, 0 CFU/ml; lanes 8, distilled water as a negative control. CFU/ml in lanes 1 and 2 were not determined because of uncountable colonies.

To determine the sensitivity, one colony suspended in LB broth with 5% (v/v) sheep blood was serially diluted 10-fold in same medium, and aliquots were used for both DNA extraction by the boiling method and counts of CFU on an LB agar plate. All 5 bands except for BACI_c47770 could be simultaneously detected from 2.99 × 10^3^ CFU/ml (Fig. 3). These amounts corresponded to 3.0 CFU/PCR reaction. The sensitivity of previously reported PCR methods including PCR using a commercial kit were less than or equal to the same serially diluted samples (data not shown).

### Application to *B*. *cereus* clinical strains genetically related to *B*. *anthracis* and field isolates

**Fig 4:**
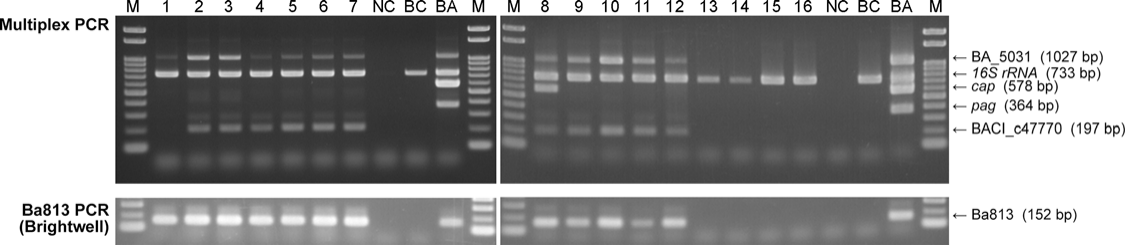
Multiplex PCR for *Bacillus* field isolates and clinical strains genetically related to *B*. *anthracis*. Field isolates in Zambia (left panel) and clinical outbreak strains in Japan (right panel), which were genetically related to *B*. *anthracis*, and field isolates in Japan (right panel) were subjected to the multiplex PCR assay developed in this study and Ba813 PCR [8]. Lane M, 100-bp DNA ladder; lane 1, *B*. *cereus* strain LZ48–5; lane 2, *B*. *cereus* strain LZ136–1; lane 3, *B*. *cereus* strain LZ136–2; lane 4, *B*. *cereus* strain LZ77–1; lane 5, *B*. *cereus* strain LZ77–2; lane 6, *B*. *cereus* strain LZ78–7; lane 7, *B*. *cereus* strain LZ78–8; lane 8, *B*. *cereus* strain GTC02891; lane 9, *B*. *cereus* strain GTC02896; lane 10, *B*. *cereus* strain GTC02916; lane 11, *B*. *cereus* strain GTC02917; lane 12, *B*. *cereus* strain GTC03222; lane 13, *B*. *cereus* strain BC_CZC1; lane 15, *B*. *cereus* strain BC_CZC2; lane 15, *B*. *pseudomycoides* strain BP_CZC1; lane 16, *B*. *pseudomycoides* strain BP_CZC2; lane NC, distilled water as a negative control; lane BC, *B*. *cereus* strain GTC02826T; lane BA, *B*. *anthracis* strain CZC5 as a positive control.

The applicability of multiplex PCR to clinical strains genetically related to *B*. *anthracis*, which were *B*. *cereus* strains isolated from outbreaks of hospital infection [23], and field isolates was studied (Table 2). Six field isolates in Zambia except for LZ48–5 and 5 clinical strains (GTC02891, GTC02896, GTC02916, GTC02917, GTC03222) were positive for both BA_5031 and BACI_c47770 (Fig. 4). Furthermore, GTC02917 was clearly *cap*-positive, being in accord with a report by Zhang *et al*. [23]. Four field isolates in Japan were 16S *rRNA*-single positive. The newly developed multiplex PCR detected each gene according to the variation of the isolates. Regional variation of isolates was not found among band patterns. Interestingly, the band patterns were closely related to genetic differences analyzed by multilocus sequence typing (MLST) defined by Priest *et al*. [29] (S3 Fig.). Basically, lineage Anthracis was both BA_5031 and 16S *rRNA*-positive, and lineage Cereus III and/or assortative lineages were BA_5031, 16S *rRNA* and BACI_c47770-positive in Clade 1. Plasmid genes (*cap* and *pag*) were detected according to isolates’ characteristics. In Clade 2, GTC02826T from lineage Tolworthi, BC_CZC1 and BC_CZC2 were 16S *rRNA*-single positive. LZ48–5 was both BA_5031 and 16S *rRNA*-positive, the pattern of which was the same as that of *B*. *anthracis* vaccine strain (Sterne 34F2); however, LZ48–5 was hemolytic (*B*. *anthracis* being non-hemolytic). In Clade 3, BP_CZC1 and BP_CZC1 were also 16S *rRNA*-single positive.

## Discussion

A rapid and sensitive method to detect *B*. *anthracis* is important in anthrax risk management and control as it enables early tracing and elimination of the infection source and timely diagnosis for appropriate treatment of the disease. Severe extra-intestinal infections due to *B*. *cereus* and *B*. *thuringiensis* in humans have recently been reported [15–18,27]. These isolates have been found to be genetically closer to *B*. *anthracis* than to *B*. *cereus* or *B*. *thuringiensis* typical strains [16,17,22], and their emergence has led to complexity of diagnosis. In this study, we developed a novel *B*. *anthracis* multiplex PCR method.

The multiplex PCR assay contains multiple internal control primers, targeting the *ACTB* on the mammalian chromosome and the *16S rRNA* on a common bacterial chromosome, to allow for monitoring of appropriate amplification. Since the actual specimens were derived from a human, mammalian animals or their products, bacteria isolates and environment samples containing commensal bacteria (e.g., water and soil), both target genes were chosen.

Universal PCR primers for amplification of bacterial *16S rRNA* are widely available [30–33]. Recently, Winsley *et al*. [34] redesigned the *16S rRNA* primer set to detect a wider range of bacteria including candidates belonging to new bacteria phyla. Since it has been assumed that their degenerate primer set hampered PCR amplification with other primers, a novel *16S rRNA* primer set (16S-663F and 16S-1395R) was designed in this study. However, the 16S-663F and 16S-1395R combination showed lower homology to *16S rRNA* among some kinds of bacteria, especially *Caldiserica* (0/1; 0%), *Chlorobi* (2/22; 9.09%), *Spirochaetes* (33/71; 46.48%) and *Thermotogae* (18/38; 47.37%) in S1 Table. These bacteria have unique morphological properties and/or differences of cultural conditions (e.g., temperature, existence of oxygen, etc.), which easily enable them to be distinguished from *B*. *anthracis*. Therefore, the *16S rRNA* primer set can detect bacterial DNA derived from cultivable bacteria in standard media and can work as an internal control without the influence of lower homology to *16S rRNA* among the above bacteria (*Caldiserica*, *Chlorobi*, *Spirochaetes* and *Thermotogae*). The *16S rRNA* primer set could also work as an internal control to examine environment samples (e.g., water and soil) because these samples contain innumerable commensal bacteria. Indeed, *16S rRNA* was amplified by the set of 16S-663F and 16S-1395R primers in all 14 bacterial DNAs including *B*. *anthracis* belonging to *Proteobacteria* and *Firmicutes* (Table 3), which were composed of major Gram-positive and-negative bacteria (data not shown). It is clear that the novel *16S rRNA* primer set works to amplify the bacterial gene and is useful for preparation of an internal control in the multiplex PCR. Recently, the Ground Anthrax Bacillus Refined Isolation (GABRI) method, which was able to detect very low level of *B*. *anthracis* from the contaminated samples (e.g., soil), has been reported [35]. The usage of GABRI method should have a better influence to the set of 16S-663F and 16S-1395R primers, since the environmental contaminants are strongly reduced by this method.

In order to detect *B*. *anthracis*, three target genes, *pag* on pXO1, *cap* on pXO2 and the Ba813 region on the chromosome, were chosen as markers of *B*. *anthracis*. Since pXO1 and pXO2 are closely related to virulence of *B*. *anthracis* strains, possession of which is valuable information to know virulence of strains. The multiplex PCR in this study could identify all *B*. *anthracis* strains, which showed variations of plasmid possession, in one reaction; however, previously reported methods could not identify them (Fig. 2). Interestingly, duplex PCR [1], single PCR to detect *cap* [10] and *pag* [7], and a commercial PCR kit could not even detect the *B*. *anthracis* genome to *B*. *anthracis* strain Sterne 34F2 clone2 (pXO1^-^/pXO2^-^/Ba813^+^/*16S rRNA* ^+^), although *B*. *anthracis*, which dose not harbor pXO1 and pXO2, is avirulent and not a public health threat (Fig. 2). Our multiplex PCR clearly enables detection of *B*. *anthracis* and its plasmid possession in one reaction. In the past decade, some *B*. *cereus* strains possessing *B*. *anthracis pag* [22] as well as *cap* [21,23] have been reported. Furthermore, the multiplex PCR could identify the characteristics of field isolates and extra-intestinal *B*. *cereus* clinical strains, especially strain GTC02891 possessing *cap* (Fig. 4), indicating that *pag* and *cap* primer sets contained in the multiplex PCR are useful for detecting *pag* and *cap* even in the genetically related strains of *B*. *anthracis*.

Although Ba813 (277 bp in length) is known as one of the important markers on the chromosome to identify and monitor *B*. *anthracis* [36], it has been reported that some *B*. *cereus* strains were found to harbor it [23,36]. In this study, the Ba813 region (1008 bp) including the 277-bp length of Ba813, tentatively termed “BA_5031”, was newly designed to amplify *B*. *anthracis* (Fig. 1). Furthermore, the hBC/BT primer set, which is capable of detecting the region, tentatively termed “BACI_c47770”, in the genetically related strains of *B*. *anthracis* (Fig. 1). These strains have been reported over the past two decades [16–18,21,23], emphasizing the importance of diagnosis and monitoring of these strains. However, there are no reports to the effective chromosomal DNA markers and assays targeting them, since the cross-reactions are frequently observed between *B*. *anthracis* and *Bacillus* strains genetically related to *B*. *anthracis* [37]. The isolates listed in Table 2 were identified by MLST (S3 Fig.). Interestingly, Zambian isolates (LZ77–1, LZ77–2, LZ78–7, LZ78–8, LZ136–1 and LZ136–2) except for LZ48–5 and clinical outbreak strains in Japan belonged to the same lineage in MLST, in which extra-intestinal *B*. *cereus* and human pathogenic *B*. *thuringiensis* strains were allocated. The band pattern of multiplex PCR also demonstrated that these strains were not typical *B*. *cereus* strains (Fig. 4); moreover, the BA_5031 and BACI_c47770 band pattern was also the same as that of *B*. *cereus* and *B*. *thuringiensis*, which are genetically closely related to *B*. *anthracis* (Ba_5031^+^/ BACI_c47770^+^) according to the result of *in silico* simulation (Fig. 1). It can be seen that our multiplex PCR discriminates *Bacillus* strains genetically related to *B*. *anthracis* as well as *B*. *anthracis* from *B*. *cereus* group species. LZ48–5 belonging to an unassigned lineage showed both a BA_5031 and *16S rRNA*-positive band pattern as did *B*. *anthracis* strain Sterne 34F2 (vaccine strain) in multiplex PCR; unfortunately, the newly developed method could not discriminate a LZ48–5 without a hemolysis test. Only in this case, the combination of multiplex PCR and a hemolysis test might be required for identification; however, *B*. *anthracis* lacking virulent plasmids is not a public health threat because strains lacking either plasmid are avirulent or significantly attenuated.

Sensitive and simple detection is required for diagnosis and treatment of anthrax, since a complicated method is burdensome for the examiner and may produce inconclusive results due to human error. Therefore, DNA extracted from liquid culture by heating was directly used in the sensitivity test to avoid a complicated method. To confirm the sensitivity in the culture condition with blood, LB broth with 5% (v/v) sheep blood was used for the test. All 5 DNA fragments could be simultaneously detected from 3.0 CFU/PCR reaction (Fig. 3). Regarding *16S rRNA* and *cap* primer sets under a multiple primer condition, specific DNA fragments were detected from 0.4 CFU/PCR reaction. The sensitivities of PCRs using a single primer set targeting *pag* and a single primer set targeting *cap* for blood samples containing *B*. *anthracis* were reported by Kurosaki *et al*. to be 3.6 and 90 CFU/PCR reaction, respectively [11]. Furthermore, the sensitivities of 6 previously reported PCR methods including PCR using a commercial kit were less than or equal to these sensitivities (data not shown). The results suggest that our multiplex PCR detected target genes equivalently to single PCR. By testing a dilution series of cultural *B*. *anthracis*, it was demonstrated that our multiplex PCR assay is a sensitive method for detection of *B*. *anthracis* as well as differentiation of *B*. *anthracis* and *B*. *anthracis*-like strains.

In conclusion, the newly developed multiplex PCR is a sensitive and practical method for detecting *B*. *anthracis* from various kinds of specimens. This assay can also discriminate the virulence of *B*. *anthracis* and its genetically related strains from other *B*. *cereus* group strains.

## Supporting Information

S1 Table Matches from the Ribosomal Database Project’s (RDP) probe match function to the primers 16S-663F and 16S-1395R designed in this study. (DOCX)

S1 Fig Specificity of *ACTB* primer set for human and animal samples. *ACTB* fragments were amplified by PCR using an *ACTB* primer set for a human sample and various kinds of animal samples. Lane M, 100-bp DNA ladder; lane NC, negative control (distilled water).
(TIF)

S2 Fig Specificity of BA_5031, *cap*, *pag*, and hBC/BT primer sets. Specific bands were amplified by single PCR each using the BA_5031 primer set (lanes 1 and 2), *cap* primer set (lanes 3 and 4), *pag* primer set (lanes 4 and 6), hBC/BT primer set (lanes 7 to 9) and *16S rRNA* primer set (lanes 10 to 12). The expected band sizes amplified by BA_5031, *cap*, *pag*, BACI_c47770 and *16S rRNA* primer sets were 1027 bp, 578 bp, 364 bp, 197 bp and 733 bp, respectively. Lane M, 100-bp DNA ladder; lanes 1, 3, 5, 7, 10, *B*. *anthracis* strain CZC5; lanes 2, 4, 6, 9, 12, distilled water. lanes 8, 11, *B*. *cereus* strain LZ77–1 as a positive control for the hBC/BT primer set. Single PCR using the *16S rRNA* primer set was used to confirm template DNA.
(TIF)

S3 Fig Phylogenetic analysis of *Bacillus* strains used in this study by multilocus sequence typing (MLST). In order to confirm the phylogenetic relationships, concatenated sequences of *B*. *cereus* type strain and 5 clinical strains, 7 field isolates in Zambia and 4 field isolates in Japan (Table 2) were used to construct a maximum likelihood tree according to the published MLST method [29]. Bold font indicates sequences from this study. The rectangles shaded with grey indicate the genetically related strains of B. anthracis in Fig. 1. Clade and lineage names are as designated by a published study [29].
(EPS)
