# Supplementary material for: A Novel Multiplex PCR Discriminates Bacillus anthracis and Its Genetically Related Strains from Other Bacillus cereus Group Species
Source: PLoS One. 2015 Mar 16;10(3):e0122004. doi: 10.1371/journal.pone.0122004 (PMC4361551; doi:10.1371/journal.pone.0122004)
Supplement: S1 Table — (DOCX) [file pone.0122004.s001.docx]

**S1 Table**

| **Primer** | **16S-663F** | **16S-1395R** | **Combination of 16S-663F and 16S-1395R** |  |
| --- | --- | --- | --- | --- |
| **Phylum** |  |  |  | **Out of Total** |
| *Actinobacteria* | 2155 | 2107 | 2032 | 2256 |
| *Aquificae* | 32 | 31 | 31 | 32 |
| *Bacteroidetes* | 699 | 759 | 681 | 782 |
| *Caldiserica* | 0 | 1 | 0 | 1 |
| *Chlamydiae* | 12 | 13 | 12 | 13 |
| *Chlorobi* | 2 | 20 | 2 | 22 |
| *Chloroflexi* | 35 | 36 | 34 | 37 |
| *Chrysiogenetes* | 4 | 4 | 4 | 4 |
| *Deferribacteres* | 12 | 12 | 12 | 12 |
| *Deinococcus-Thermus* | 73 | 72 | 72 | 73 |
| *Dictyoglomi* | 5 | 5 | 5 | 5 |
| *Elusimicrobia* | 1 | 1 | 1 | 1 |
| *Fibrobacteres* | 3 | 3 | 3 | 3 |
| *Fusobacteria* | 36 | 50 | 34 | 54 |
| *Gammatimonadetes* | 1 | 1 | 1 | 1 |
| *Lentisphaerae* | 2 | 2 | 2 | 2 |
| *Nitrospira* | 8 | 8 | 8 | 8 |
| *Planctomycetes* | 9 | 11 | 9 | 11 |
| *Proteobacteria* | 3106 | 3239 | 2992 | 3378 |
| *Spirochaetes* | 35 | 62 | 33 | 71 |
| *Synergistetes* | 21 | 18 | 18 | 21 |
| *Tenericutes* | 182 | 180 | 176 | 186 |
| *Thermodesulfobacteria* | 7 | 7 | 7 | 7 |
| *Thermotogae* | 26 | 30 | 18 | 38 |
| BRC1 | 0 | 0 | 0 | 0 |
| OD1 | 0 | 0 | 0 | 0 |
| OP11 | 0 | 0 | 0 | 0 |
| SR1 | 0 | 0 | 0 | 0 |
| TM7 | 0 | 0 | 0 | 0 |
| WS3 | 0 | 0 | 0 | 0 |
| *Armatimonadetes* | 2 | 2 | 2 | 2 |
| *Verrucomicrobia* | 41 | 40 | 40 | 41 |
| *Acidobacteria* | 14 | 14 | 14 | 14 |
| *Firmicutes* | 1960 | 1915 | 1914 | 1964 |
| *Cyanobacteria/Chloroplast* | 148 | 167 | 145 | 170 |
| **domain *Bacteria* Total** | 8631 | 8810 | 8302 | 9209 |
| **Percentage** | 93.72 | 95.67 | 90.15 | 100 |
