# Supplementary figures and images for: A Novel Multiplex PCR Discriminates Bacillus anthracis and Its Genetically Related Strains from Other Bacillus cereus Group Species
Source: PLoS One. 2015 Mar 16;10(3):e0122004. doi: 10.1371/journal.pone.0122004 (PMC4361551; doi:10.1371/journal.pone.0122004)

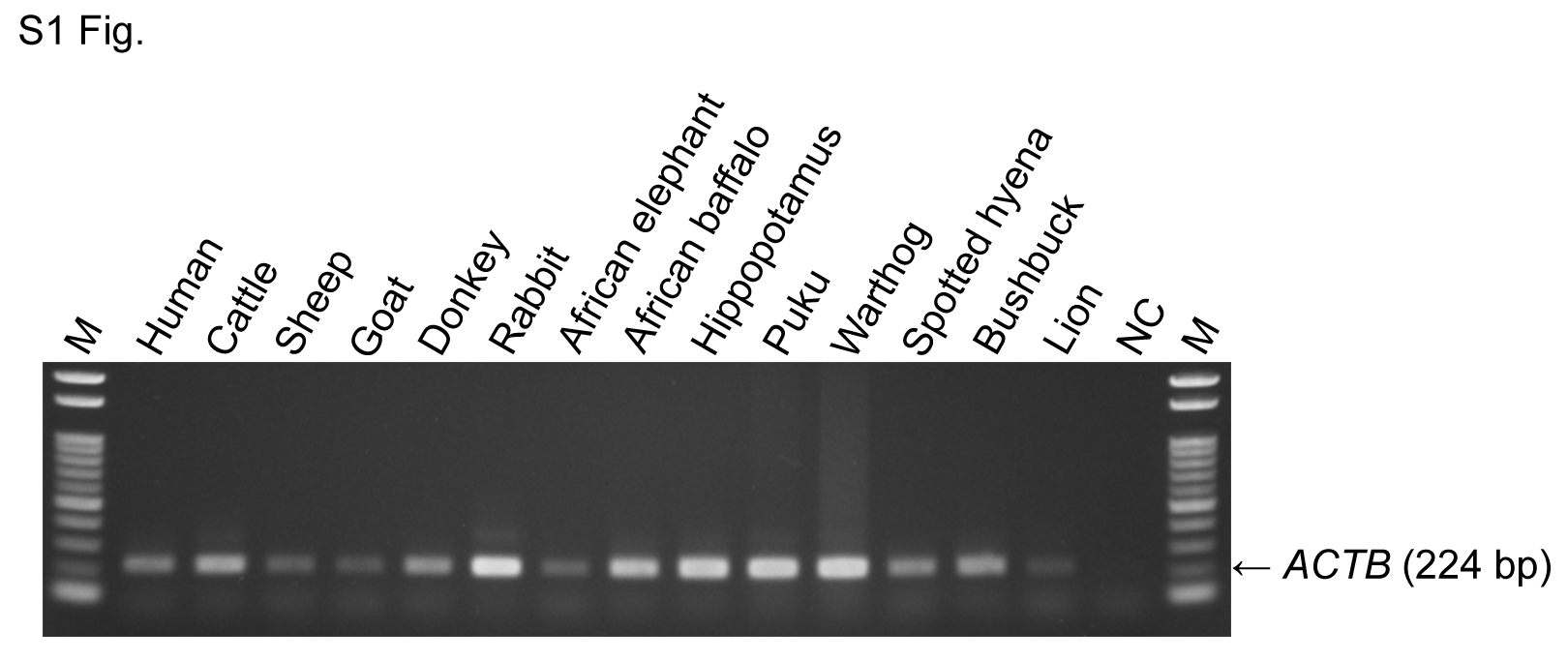

Supplement: S1 Fig — ACTB fragments were amplified by PCR using an ACTB primer set for a human sample and various kinds of animal samples. Lane M, 100-bp DNA ladder; lane NC, negative control (distilled water). (TIF) [file pone.0122004.s002.tif]

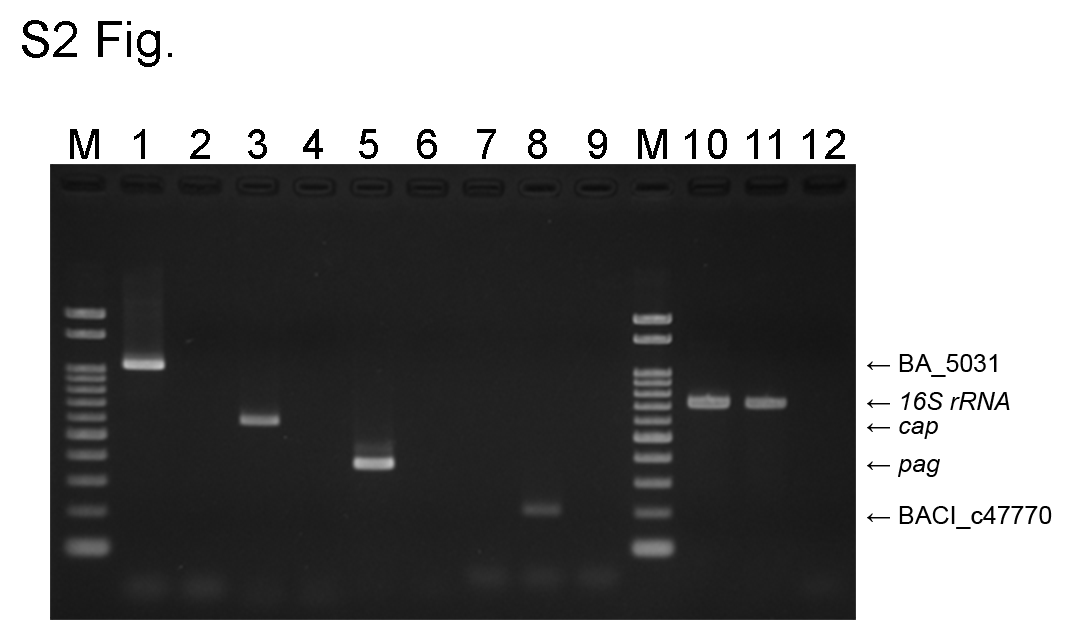

Supplement: S2 Fig — Specific bands were amplified by single PCR each using the BA_5031 primer set (lanes 1 and 2), cap primer set (lanes 3 and 4), pag primer set (lanes 4 and 6), hBC/BT primer set (lanes 7 to 9) and 16S rRNA primer set (lanes 10 to 12). The expected band sizes amplified by BA_5031, cap, pag, BACI_c47770 and 16S rRNA primer sets were 1027 bp, 578 bp, 364 bp, 197 bp and 733 bp, respectively. Lane M, 100-bp DNA ladder; lanes 1, 3, 5, 7, 10, B. anthracis strain CZC5; lanes 2, 4, 6, 9, 12, distilled water. lanes 8, 11, B. cereus strain LZ77–1 as a positive control for the hBC/BT primer set. Single PCR using the 16S rRNA primer set was used to confirm template DNA. (TIF) [file pone.0122004.s003.tif]
